# Role of Zinc in Mucosal Health and Disease: A Review of Physiological, Biochemical, and Molecular Processes

**DOI:** 10.7759/cureus.8197

**Published:** 2020-05-19

**Authors:** Abbas Hassan, Kahmalia-Kalee Sada, Suvetha Ketheeswaran, Arun Kumar Dubey, Malpe Surekha Bhat

**Affiliations:** 1 Plastic and Reconstructive Surgery, Northwestern University Feinberg School of Medicine, Chicago, USA; 2 Internal Medicine, Xavier University School of Medicine, Oranjestad, ABW; 3 Surgery, Xavier University School of Medicine, Oranjestad, ABW; 4 Pharmacology, Xavier University School of Medicine, Oranjestad, ABW; 5 Biochemistry and Genetics, American University School of Medicine, Oranjestad, ABW

**Keywords:** zinc, zinc deficiency, zinc balance, mucosal health

## Abstract

Zinc is an essential trace element of all highly proliferating cells in the human body. It is essential to the development and growth of all organisms. Zinc plays a critical role in modulating resistance to infectious agents and reduces the duration, severity, and risk of diarrheal disease via improved regeneration of intestinal epithelium, improved absorption of water and electrolytes, increased levels of brush border enzymes, and, possibly, an enhancement in the immune response allowing better clearance of pathogens. On the cellular level, zinc finger motifs play various roles including diverse functions that involve specific gene expression for ion channels throughout the body. It maintains the function and the structure of the membrane barrier by contributing to host defense, which is particularly crucial in the intestines due to the continuous exposure to noxious agents and pathogens. Zinc deficiency is characterized by impaired immune function, loss of appetite, and growth retardation. More severe cases cause diarrhea, delayed sexual maturation, hair loss, eye and skin lesions, impotence and hypogonadism in males, as well as weight loss, taste abnormalities, delayed healing of wounds, and mental lethargy. The objective of this study is a critical review of the molecular and genetic regulation of zinc in various cellular processes and organs, the association between zinc and diarrheal disease, the recommended dietary zinc intake, and the effects of zinc deficiency on the human body.

## Introduction and background

Diarrheal diseases are a significant cause of morbidity and mortality in developing countries. It is estimated that diarrheal diseases cause about 1000 million episodes and >3 million deaths annually worldwide [1].

It is one of the most common illnesses of children and a major cause of childhood and infant mortality in developing countries. Malnutrition plays a major role in the etiology, prognosis, and management of persistent diarrhea in children [2]. The mechanisms that contribute to malnutrition in diarrheal diseases include loss of appetite, the withholding of food, direct loss of nutrients in the stool, intestinal malabsorption, the catabolic effect, and the inappropriate use of certain foods in treating the patient [3].

A deficiency of several specific nutrients has been implicated. Zinc is notable among these nutrients. An association between diarrhea and the abnormalities of zinc has been implicated in several studies. These abnormalities include a negative zinc balance, increased fecal zinc losses, low tissue zinc concentrations, and hypozincemia [4]. The increased loss of zinc in the stool can affect the duration and severity of the diarrheal disease, thus contributing to a vicious circle.

The mechanism by which zinc causes diarrhea has not been clearly elucidated. The objective of this study is a critical review of the molecular and genetic regulation of zinc in various cellular processes and organs, the association between zinc and diarrheal disease, the recommended dietary zinc intake, and the effects of zinc deficiency on the human body.

## Review

Methods

We searched the PubMed, Medline, Embase, and Cochrane databases for studies that met our eligibility criteria. The most recent search was performed in January 2020 using the following terms in different combinations: “zinc,” “biomembranes,” “immunity,” “motifs,” and “deficiency.” The selection and review process was done manually in a randomized and blinded fashion between three independent reviewers. Also reviewed were the bibliographies of the studies that met the eligibility criteria for the review, including papers that were not present in our initial search. Eligibility criteria included peer-reviewed studies discussing the biochemical, physiological, and molecular roles of zinc in mucosal health and disease in the English language. Forty-five total studies were included in the final selection.

Physiological effects of zinc

Zinc is an essential element for all highly proliferating cells in the human body. Zinc is essential to the development and growth of all organisms. Zinc is present in all organs, tissues, fluids, and secretions of the body. The human body contains about 2-4 grams of zinc, with only 12-16 μmol/L occurring in the plasma [5]. Zinc also plays a role in all six classes of enzymes, as well as replication and transcription factors [6-7].

Zinc plays a critical role in modulating resistance to infectious agents and reduces the duration, severity, and risk of diarrheal disease. Zinc stimulates mucosal and serosal ion absorption after the addition to epithelial monolayers. Other effects of zinc include improved regeneration of intestinal epithelium, improved absorption of water and electrolytes, increased levels of brush border enzymes, and, possibly, an enhancement in the immune response, allowing better clearance of pathogens.

Its importance is emphasized in conditions of zinc deficiency, when the brain, immune system, and gut are impaired.

Effects of zinc on intestinal ion transport

The absorptive effects after the addition of zinc explain the mechanism of severe diarrhea associated with zinc deficiency. Several studies have shown that diarrhea is more severe in zinc-deficient subjects [8]. The results of one study have indicated that net sodium and water transport from the large and small intestine was significantly decreased in zinc-deficient rats when compared to pair-fed controls and for ad libitum-fed rats [8]. The addition of zinc to the luminal side or the basolateral side of the enterocyte induces a chloride-dependent, dose-related decrease in short-circuit current indicating ion absorption [9].

The effects of zinc on intestinal ion transport have not been elucidated; recent studies investigated the effects of zinc on sodium (Na) absorption and chloride (Cl) secretion to determine whether zinc inhibits Cl secretion or enhances Na absorption.

Zinc was found to inhibit Cyclic adenosine monophosphate (cAMP)-induced chloride-dependent fluid secretion by inhibiting basolateral potassium (K) channels. However, this inhibition was shown to be specific to cAMP-activated K channels and not calcium-mediated K channels [10].

Immune-modulating effects of zinc

Zinc is essential for facilitating the coordination of the immune activation during responses to infection. Recent studies have reported that zinc deficiency increases vital organ damage, systemic inflammation, and mortality in a small animal model of sepsis [11-12]. Altered zinc levels can disturb the functions of the immune system. It affects the recruitment of neutrophils and chemotaxis because zinc directly induces chemotactic activity in polymorphonuclear (PMN) leukocytes [13]. In cases of zinc deficiency, the number of granulocytes was shown to be decreased [14]. Natural killer cell activity, generation of oxidative burst, and phagocytosis of neutrophils and macrophages are impaired by decreased zinc levels [15]. Zinc also affects the levels of various cytokines that are modulators within the immune system. Studies have shown that when peripheral blood mononuclear cells are incubated with zinc, interleukin (IL)-1, IL-6, tumor necrosis factor (TNF)-α, soluble (s) IL-2 receptor, and interferon-gamma (IFN-γ) are released [16-17]. The beneficial effects of zinc in infectious diarrhea can be explained by both the immune-modulating effects of zinc and the reduction in ion secretion. It was shown that the release of TNF-α after stimulation with zinc is not caused by enhanced translation of already expressed messenger ribonucleic acid (mRNA) but rather a de novo transcription of mRNA [18]. A recent study showed that zinc results in a substantial reduction in cholera toxin (CT)-induced ion secretion and cAMP concentration. However, E. coli heat-stable (ST)-induced ion secretion and cyclic guanosine monophosphate (cGMP) concentration were not affected [9].

Zinc finger motifs-associated transcription of ion channels

Regulation and Transcription of Genes

Zinc finger (ZnF) motifs play various roles at the cellular level and include diverse functions that involve specific gene expression for ion channels throughout the body. Zinc finger proteins are involved in recognizing deoxyribonucleic acid (DNA), transcription, and protein folding. The identification of transcriptional factors with the ability to recognize and bind to specific DNA sequences has provided essential clues to elucidating these mechanisms. Zinc is critical for the activities of various enzymes that contribute to cellular signaling pathways as well as transcription factors. It is believed to maintain the structures of proteins and/or serve as a cofactor in these proteins by binding tightly to various zinc-binding motifs, including zinc finger, ring finger, and LIM domains [19]. The role of zinc has been observed in various experiments, which have shown a direct correlation with zinc in critical pathways and cell signaling that involve vast tasks such as differentiation of tissues, hormonal secretion, effects on receptors, and channels. The manner that zinc fingers are able to achieve its physiological effects in the body is through the modification and expression of genes. Zinc finger motifs have an influential role in the upregulation and downregulation of genes through DNA binding and transcription factors that are necessary for increasing the number of ion channels, proteins, and receptors and for the stabilization of membranes. Although initially recognized in an RNA polymerase III transcription factor, recent analysis has revealed potential metal-binding fingers as a surprisingly common structure in a variety of nucleic acid-binding proteins, including factors likely to influence polymerase II transcription [20]. Zinc finger motifs allow for the binding of specific DNA proteins, for example, transcription factors that are essential for the normal function of cells, cellular differentiation, growth, and maintenance of multiple organs. The presence of zinc in the body allows for certain biochemical processes and reactions to occur during metabolism to carry out necessary bodily functions. Zinc serves as a cofactor for metalloenzymes, which are essential in the process of nucleic acid metabolism. Zinc can also be found as a component of DNA binding proteins, which contain zinc fingers and other structural characteristics. Zinc is an essential component of the diet for protein synthesis and gene expression that are necessary for reactions to take place in the body. Cellular metabolism requires zinc to assist in functions such as DNA synthesis, transcription, and translation for the production of specific proteins. Any significant deficiency in Zinc can cause an interruption in important cellular processes and lead to pathologies.

Effects of Motifs on Ion Channels in the Gastrointestinal System

Zinc finger motifs have been shown to increase gene expression for specific receptors, channels, and transportation throughout the body. Zinc can incorporate itself into small areas within the cell plasma membrane and manipulate structures for transportation to occur. The effects of zinc embedded in the cell plasma membrane is the passage of ions, signals, binding sites, and enzymatic activity. Zinc can assist in passive transportation through mechanisms that are not clear. The rate and route of zinc transport are primarily dependent on the chemical forms of zinc presented to the cell. It is generally believed that the pores, channels, and carriers involved in zinc transport are not highly selective. The zinc that is continuously present in these structures may be significant in terms of zinc mass and regulatory function in ion transport systems [21]. Observing the effects of individuals who have zinc deficiencies, one of the common issues is diarrhea. Zinc plays a significant role in gene expression for various receptors, signaling pathways, as well as ion channels found throughout the gastrointestinal system that assists with proper digestion and function. Zinc assists with the maintenance of water distribution leaving and entering the cell following ions and their concentration. Zinc can make metabolic alterations at a cellular level to inhibit cyclic adenylate monophosphate-stimulated chloride secretion throughout the gastrointestinal system. This is achieved through blocking K+ channels located in places like the ileum. Once zinc inhibits the basolateral potassium channels, this indirectly prevents cyclic adenylate monophosphate from stimulating chloride channels. Chloride is prevented from being secreted, which prevents excessive water from being dragged into the lumen, which would cause diarrhea. Ion concentration and homeostasis occur within the lumen of the gut due to a property that can drag excess water into the lumen with sodium, which may result in diarrhea. Zinc has a protective property against this and is an essential component to achieving a healthy and fully functioning gut. As mentioned earlier, the research that was done on rats found that zinc has the capacity to inhibit cAMP-induced chloride secretion. In the small intestines, the ileum contains potassium channels where Zinc exerts its effect to inhibit these cAMP-activated channels preventing the secretion of chloride. Zinc can modulate cell signaling and affects the complex process that affects how the intestines function with ions. The mechanisms suggested for this modulation fall into three categories: (1) blockage of receptor-gated and voltage-gated ion channels, (2) regulation of protein and phosphatidylinositol phosphorylation and dephosphorylation, and (3) regulation of hormone binding to cell surface receptors [22-23]. Manifestations found in patients with moderate to severe zinc deficiency have been explained by identifying many functions and genes expressed with the assistance of zinc. Phenotypic expression of the rare autosomal recessive disorder, acrodermatitis enteropathica (AE), was found to be due to defects in zinc metabolism. Mutations in ZIP4 were found to be causative of AE. ZIP4 encodes a member of the Zrt-Irt-like protein (ZIP)/SLC39 zinc transporter family that is expressed in intestinal organs, indicating that the intestinal absorption of zinc is a critical process [19].

Zinc and the formation and stabilization of biomembranes

Zinc plays an essential role in critical areas of metabolism and is required by a variety of enzymes. Thus, cells have evolved mechanisms for maintaining zinc homeostasis when available supplies decrease. In addition, cells have evolved some mechanisms to adapt to suboptimal levels of zinc [24]. Zinc maintains the function and structure of the membrane barrier by contributing to host defense, which is particularly crucial in the intestines due to the continuous exposure to noxious agents and pathogens [25-27]. The intestinal epithelium constitutes a barrier that involves intercellular junctional complexes and provides a continuous seal between neighboring cells [28]. Several studies have shown that zinc deficiency causes ulcerations of the small intestines, alters the membrane barrier permeability, and enhances the degradation of several intracellular complexes [25,29-30].

Interestingly, a reduced level of mucosal zinc has been shown in patients with chronic intestinal permeability disturbances [31]. Previous studies have also shown that zinc deficiency is associated with inflammatory cell infiltration that causes damage to the gut membrane [29-30]. A recent study provided new evidence on the role of zinc in the maintenance of membrane barrier integrity and prevention of massive neutrophil infiltration, showing that zinc deficiency impairs the integrity of the apical junction complexes, membrane permeability, and the cytoskeleton of intestinal cells, favoring PMN leukocytes migration through the paracellular space [32].

Zinc dietary requirements

A 70 kg adult contains about 2-3 g zinc, about 0.1% of which are replenished daily. The daily amount of zinc that is needed is relatively small, about 2-3 mg in adults to compensate for the relatively small loss of zinc in urine, stool, and sweat [33-34]. Intake recommendations for zinc and other nutrients are provided in the Dietary Reference Intakes (DRIs) developed by the Food and Nutrition Board (FNB) at the Institute of Medicine of the National Academies (formerly National Academy of Sciences) [35]. Recommendations are stratified for age, gender, and conditions of high metabolic needs such as lactation and pregnancy. For example, lower values are given for younger individuals whereas higher values, at least at 50% higher, are given to vegetarians because zinc is not readily available from a vegetarian diet [36]. Zinc requirements for nursing and pregnant woman are also higher. In this population, it's recommended to increase the daily intake by 3 and 4 mg respectively. However, current recommendations do not consider the effects of diets rich in inhibitors of zinc absorption, a factor to increase the recommended doses in healthy adults. The current average daily level of intake sufficient to meet the nutrient requirements of nearly all (97%-98%) healthy individuals (Recommended Dietary Allowances) for zinc are listed in Table 1.

**Table 1:** Recommended Dietary Allowances (RDA) for Zinc * Adequate Intake (AI) Reference [37]

Age	Female	Male	Pregnancy	Lactation
0–6 months	2 mg*	2 mg*		
7–12 months	3 mg	3 mg		
1–3 years	3 mg	3 mg		
4–8 years	5 mg	5 mg		
9–13 years	8 mg	8 mg		
14–18 years	9 mg	11 mg	12 mg	13 mg
19+ years	8 mg	11 mg	11 mg	12 mg

Effects of zinc deficiency on the human body

Overview

A zinc deficiency is characterized by impaired immune function, loss of appetite, and growth retardation. More severe cases of zinc deficiency cause diarrhea, delayed sexual maturation, hair loss, eye and skin lesions, impotence, and hypogonadism in males [38]. Weight loss, taste abnormalities, delayed healing of wounds, and lethargy can also occur [39]. The most commonly used indices for evaluating zinc deficiency is serum or plasma zinc levels, but due to the tight hemostatic control mechanisms, these levels do not necessarily reflect cellular zinc status [5]. Thus, clinicians must consider the risk factors and symptoms of zinc deficiency when determining the need for zinc supplementation [35].

Wound Healing

Zinc plays a role as a cofactor in numerous transcription factors and enzyme systems, including keratinocyte migration during wound repair and zinc-dependent metalloproteinases that augment auto debridement. Zinc also confers resistance to epithelial apoptosis through cytoprotection by the antioxidant activity of the cysteine-rich metallothionines [37]. Zinc deficiency can lead to delayed wound healing and pathological changes. Studies have shown that patients with chronic leg ulcers have low serum zinc levels and abnormal zinc metabolism [40]. The authors of another study have concluded that supplementing zinc sulfate might be effective for some patients who have low serum zinc levels.

Diarrhea

Zinc deficiency causes alterations in the body’s immune responses to infections that increase susceptibility to infection, such as those causing diarrhea [41]. Studies have shown that after taking zinc supplements, malnourished children in Southeast Asia, South America, Africa, and India experience shorter courses of infectious diarrhea. The dose of zinc received in these studies is 4-40mg a day in the form of zinc sulfate, zinc acetate, and zinc gluconate [42]. Several randomized controlled trials and meta-analysis studies have shown that zinc helps reduce the duration and severity of diarrhea in malnourished or zinc-deficient children [43]. However, the effects of supplementing zinc to children with adequate zinc status with diarrhea are not apparent. The UNICEF and the World Health Organization now recommend short-term zinc supplementation (20 mg of zinc per day or 10 mg for infants under 10-14 days) to treat acute childhood diarrhea.

Immune System Function

As discussed earlier, zinc plays an essential role in the modulation of the immune system. Zinc deficiency impairs neutrophil and macrophage action, natural killer cell activity, and complement activity [44]. Individuals with zinc deficiency have also been shown to have reduced lymphocytes proliferation response to mitogens and other alterations that are corrected by supplementing zinc [45]. The effects of zinc deficiency on the immune system function help explain the increased susceptibility to pneumonia and other infections in children with low zinc status in developing countries [46].

## Conclusions

Zinc is an essential element of all highly proliferating cells in the human body. It plays a critical role in modulating resistance to infectious agents and reduces the duration, severity, and risk of diarrheal disease. Zinc deficiency is characterized by impaired immune function, loss of appetite, and growth retardation. This study provided an overview of the molecular and genetic regulation of zinc in various cellular processes and organs, the association between zinc and diarrheal disease, the recommended dietary zinc intake, and the effects of zinc deficiency on the human body.

## References

[1] Bern C, Martines J, De Zoysa I, Glass RI (1992). The magnitude of the global problem of diarrhoeal disease: a ten-year update. Bull World Health Organ.

[2] Hambidge KM (1992). Zinc and diarrhea. Acta Paediatr.

[3] Chen LC, Scrimshaw NS, Kimati VP (1982). Diarrhea and malnutrition: interactions, mechanisms, and interventions. Food Nutr Bull.

[4] Wolman SL, Anderson CH, Marliss EB, Jeejeebhoy KN (1979). Zinc in total parenteral nutrition: requirements and metabolic effects. Gastroenterology.

[5] Mills CF (1898). Zinc in Human Biology. Zinc in human biology..

[6] Auld DS (2001). Zinc coordination sphere in biochemical zinc sites. Biometals.

[7] Hassan AM, Bhat S, Prasad VN (2017). Inhaled nitric oxide in treatment of persistent pulmonary hypertension of neonate: an insight into biochemical pathways. Asian J Med Health.

[8] Ghishan FK (1984). Transport of electrolytes, water, and glucose in zinc deficiency. J Pediatr Gastroenterol Nutr.

[9] Canani R, Cirillo P, Buccigrossi V (2005). Zinc inhibits cholera toxin-induced, but not Escherichia coli heat-stable enterotoxin-induced, ion secretion in human enterocytes. J Infect Dis.

[10] Hoque KM, Rajendran VM, Binder HJ (2005). Zinc inhibits cAMP-stimulated Cl secretion via basolateral K-channel blockade in rat ileum. Am J Physiol Gastrointest Liver Physiol.

[11] Knoell DL, Julian MW, Bao S (2009). Zinc deficiency increases organ damage and mortality in a murine model of polymicrobial sepsis. Crit Care Med.

[12] Bao S, Liu MJ, Lee B, Besecker B, Lai JP, Guttridge DC, Knoell DL (2010). Zinc modulates the innate immune response in vivo to polymicrobial sepsis through regulation of NF-κB. Am J Physiol Lung Cell Mol Physiol.

[13] Hujanen ES, Seppä ST, Virtanen K (1995). Polymorphonuclear leukocyte chemotaxis induced by zinc, copper and nickel in vitro. Biochim Biophys Acta.

[14] Prasad AS (2000). Effects of zinc deficiency on immune functions. J Trace Elem Med Biol.

[15] Keen CL, Gershwin ME (1990). Zinc deficiency and immune function. Annu Rev Nutr.

[16] Driessen C, Hirv K, Rink L, Kirchner H (1994). Induction of cytokines by zinc ions in human peripheral blood mononuclear cells and separated monocytes. Lymphokine Cytokine Res.

[17] Salas M, Kirchner H (1987). Induction of interferon-γ in human leukocyte cultures stimulated by Zn2+. Clin Immunol Immunopathol.

[18] Wellinghausen N, Fischer A, Kirchner H, Rink L (1996). Interaction of zinc ions with human peripheral blood mononuclear cells. Cell Immunol.

[19] Murakami M, Hirano T (2008). Intracellular zinc homeostasis and zinc signaling. Cancer Sci.

[20] Evans RM, Hollenbergt SM (1988). Zinc fingers: gilt by association. Cell.

[21] Bettger WJ, O'Dell BL (1993). Physiological roles of zinc in the plasma membrane of mammalian cells. J Nutr Biochem.

[22] Bashford CL, Alder GM, Graham JM, Menestrina G, Pasternak CA (1988). Ion modulation of membrane permeability: effect of cations on intact cells and on cells and phospholipid bilayers treated with pore-forming agents. J Membr Biol.

[23] Hassan AM, Prasad VN, Fidelis N (2019). Drugs used in thromboembolic disorders: an insight into their mechanisms. Asian J Cardiol Res.

[24] Eide DJ (2009). Homeostatic and adaptive responses to zinc deficiency in Saccharomyces cerevisiae. J Biol Chem.

[25] Sturniolo GC, Di Leo V, Ferronato A, D’odorico A, D’Incà R (2001). Zinc supplementation tightens “leaky gut” in Crohn’s disease. Inflamm Bowel Dis.

[26] Bao S, Knoell DL (2006). Zinc modulates cytokine-induced lung epithelial cell barrier permeability. Am J Physiol Lung Cell Mol Physiol.

[27] Hassan A, Dubey AK, Bhat MS (2019). Pyridoxine: the ‘Ba.Six’ of use in nausea and vomiting of pregnancy. J Clin Diagn Res.

[28] Rodriguez P, Darmon N, Chappuis P, Candalh C, Blaton MA, Bouchaud C, Heyman M (1996). Intestinal paracellular permeability during malnutrition in guinea pigs: effect of high dietary zinc. Gut.

[29] Furuse M, Itoh M, Hirase T, Nagafuchi A, Yonemura S, Tsukita S (1994). Direct association of occludin with ZO-1 and its possible involvement in the localization of occludin at tight junctions. J Cell Biol.

[30] Mengheri E, Nobili F, Vignolini F, Pesenti M, Brandi G, Biavati B (1999). Bifidobacterium animalis protects intestine from damage induced by zinc deficiency in rats. J Nutr.

[31] Canali R, Vignolini F, Nobili F, Mengheri E (2000). Reduction of oxidative stress and cytokine-induced neutrophil chemoattractant (CINC) expression by red wine polyphenols in zinc deficiency induced intestinal damage of rat. Free Radic Biol Med.

[32] Sturniolo GC, Mestriner C, Lecis PE (1998). Altered plasma and mucosal concentrations of trace elements and antioxidants in active ulcerative colitis. Scand J Gastroenterol.

[33] Finamore A, Massimi M, Conti Devirgiliis L, Mengheri E (2008). Zinc deficiency induces membrane barrier damage and increases neutrophil transmigration in Caco-2 cells. J Nutr.

[34] Wastney ME, Aamodt RL, Rumble WF, Henkin RI (1986). Kinetic analysis of zinc metabolism and its regulation in normal humans. Am J Physiol.

[35] Sandstead HH, Smith Jr JC (1996). Deliberations and evaluations of approaches, endpoints and paradigms for determining zinc dietary recommendations. J Nutr.

[36] Lupton JR, Brooks J, Butte N, Caballero B, Flatt J, Fried S (Washington, DC). Dietary Reference Intakes for Energy, Carbohydrate, Fiber, Fat, Fatty Acids, Cholesterol, Protein, and Amino Acids. Institute of Medicine.

[37] Maret W, Sandstead HH (2006). Zinc requirements and the risks and benefits of zinc supplementation. J Trace Elem Med Biol.

[38] Prasad AS (2004). Zinc deficiency: its characterization and treatment. Met Ions Biol Syst.

[39] Wang LC, Busbey S (2005). Acquired acrodermatitis enteropathica. N Engl J Med.

[40] Lansdown AB, Mirastschijski U, Stubbs N, Scanlon E, Ågren MS (2007). Zinc in wound healing: theoretical, experimental, and clinical aspects. Wound Repair Regen.

[41] Wilkinson EAJ, Hawke CI (1998). Does oral zinc aid the healing of chronic leg ulcers? A systematic literature review. Arch Dermatol.

[42] Black RE (1998). Therapeutic and preventive effects of zinc on serious childhood infectious diseases in developing countries. Am J Clin Nutr.

[43] Lukacik M, Thomas RL, Aranda JV (2008). A meta-analysis of the effects of oral zinc in the treatment of acute and persistent diarrhea. Pediatrics.

[44] Wintergerst ES, Maggini S, Hornig DH (2007). Contribution of selected vitamins and trace elements to immune function. Ann Nutr Metab.

[45] Beck F, Prasad A, Kaplan J, Fitzgerald J, Brewer G (1997). Changes in cytokine production and T cell subpopulations in experimentally induced zinc-deficient humans. Am J Physiol.

[46] Brooks WA, Santosham M, Naheed A (2005). Effect of weekly zinc supplements on incidence of pneumonia and diarrhoea in children younger than 2 years in an urban, low-income population in Bangladesh: randomised controlled trial. Lancet.

